# Poly(3-Methylthiophene) Thin Films Deposited Electrochemically on QCMs for the Sensing of Volatile Organic Compounds

**DOI:** 10.3390/s16040423

**Published:** 2016-03-23

**Authors:** Sadullah Öztürk, Arif Kösemen, Zafer Şen, Necmettin Kılınç, Mika Harbeck

**Affiliations:** 1 Faculty of Engineering, Fatih Sultan Mehmet Vakif University, Istanbul 34080, Turkey; 2 Department of Physics, Mus Alparslan University, Mus 49100, Turkey; akosemen@gtu.edu.tr; 3 Science Faculty, Department of Physics, Gebze Technical University, Kocaeli 41400, Turkey; 4 Materials Institute, TUBITAK Marmara Research Center, Gebze 41470, Turkey; zafer.sen@tubitak.gov.tr (Z.S.); mika.harbeck@tubitak.gov.tr (M.H.); 5 Mechatronics Engineering Department and Nanotechnology Application & Research Center, Nigde University, Nigde 51245, Turkey; nkilinc@nigde.edu.tr

**Keywords:** chemical gas sensor, poly(3-methylthiophene), humid air, electrochemical deposition, QCM, VOCs, DMMP

## Abstract

Poly(3-methylthiophene) (PMeT) thin films were electrochemically deposited on quartz crystal microbalance QCM transducers to investigate their volatile organic compound (VOC) sensing properties depending on ambient conditions. Twelve different VOCs including alcohols, ketones, chlorinated compounds, amines, and the organosphosphate dimethyl methylphosphonate (DMMP) were used as analytes. The responses of the chemical sensors against DMMP were the highest among the tested analytes; thus, fabricated chemical sensors based on PMeT can be evaluated as potential candidates for selectively detecting DMMP. Generally, detection limits in the low ppm range could be achieved. The gas sensing measurements were recorded at various humid air conditions to investigate the effects of the humidity on the gas sensing properties. The sensing performance of the chemical sensors was slightly reduced in the presence of humidity in ambient conditions. While a decrease in sensitivity was observed for humidity levels up to 50% r.h., the sensitivity was nearly unaffected for higher humidity levels and a reliable detection of the VOCs and DMMP was possible with detection limits in the low ppm range.

## 1. Introduction

Modernization efforts are evaluated as a main factor in attaining an easy and inhabitable life, although at the same time they lead to a rapidly increasing destruction of our environment and atmosphere. Major factors of environmental pollution are the use of fossil fuels to supply energy demands as well as the use of volatile organic compounds (VOCs) in the field of manufacturing. VOCs are known to give rise to several diseases including asthma, dizzies, and headache during short-term exposure and to possibly increase risk of various cancer diseases in the long term. They can combine with inhaled air via evaporation or sublimation with low boiling points [1,2].

The monitoring and controlling of VOC contaminants in any ambient condition is crucial to protecting our environment, atmosphere, and health in order to leave an inhabitable world for our future generations as a heritage. Gas chromatography, mass spectroscopy, and Fourier transform infrared spectroscopy are very powerful, reliable systems with high accuracy for detecting VOCs, albeit known to be expensive, time-consuming, demanding in their sampling requirements, and requiring trained operators [3,4]. In order to overcome these challenges, chemical gas sensors have been developed depending on various chemical and physical approximations [5]. Commonly, chemical gas sensors are composed of two main parts: Firstly, the sensitive layer is made of metals, metal oxides, organic or organometallic compounds or polymers, interacting with the gas molecules in ambient conditions and then changing its physical properties upon the chemical interaction such as resistance, capacitance, ion mobility, absorbance, *etc.* Secondly, the transducing element turns these changes into a readable signal and allows for a reading of these changes [5,6]. A quartz crystal microbalance (QCM) based on the piezoelectric effect of the quartz crystal is one of the transducing elements widely used in chemical gas detection or biosensor applications [7]. When a suitable periodic voltage is applied to the electrodes of the piezoelectric material, they start to emit a certain vibration frequency known as the resonance frequency. The frequency shifts upon adsorbing gas molecules on the surface depending on mass load caused by the gas molecules. This frequency decrease is evaluated as the sensor signal. In sensor applications, several performance parameters have been defined as sensitivity, selectivity, reliability, long-term stability, *etc.* [8,9]. Thus, characterization of the sensitive materials has gained great attention to satisfy expectations. Humidity in the air is a very important parameter in gas sensing applications and needs to be considered to fabricate and investigate more suitable sensor devices to be operated under real-world conditions. Bachar *et al.* investigated real-world VOCs sensing properties of polycyclic aromatic hydrocarbons deposited on chemiresistive and QCM-based transducers depending on humidity conditions. The QCM-based sensors were more stable than the chemiresistors in humid air [10]. Zilberman *et al.* enhanced the sensing properties of carbon nanotubes for detecting nonpolar volatile organic compounds. The sensing of the volatile organic compounds in the breath could be used as noninvasive diagnostic methods for cancers [11].

As a member of the polymer family, conducting polymers including polyacetylene, polyaniline, polypyrrole, polythiophene, and their derivatives are very good candidates and sensitive materials for gas sensing applications due to their low-cost and simple fabrication techniques and alterable electrical properties using, for example, doping with foreign materials [12,13,14,15,16,17,18]. For instance, Si *et al.* coated different polymers on the QCM transducers via various deposition techniques to investigate VOCs sensing properties [4]. Major successes of the QCM transducers compared to other types of gas sensors are, for example, low operation temperature, fast response and recovery times, eliminating short-cut circuit problem between electrodes, and lack of complexity in the design of the electrodes. QCM-based sensors modified by polycyclic hydrocarbons and derivatives were tested in their responses to VOCs under varying humidity conditions. These materials are very suitable for discrimination of the target analytes because the sensing properties of the polycyclic hydrocarbons were directly linked to the shape and size and thus did not require complicated pattern recognition systems [19].

In this work, poly(3-methylthiophene) thin films were coated on QCM transducers via electrochemical deposition techniques under various deposition conditions. The fabricated chemical sensor elements were tested in their responses to various VOCs and the organophoshate dimethyl methylphosponate (DMMP) at room temperature to investigate their gas sensing properties. The effects of the deposition condition on the sensor responses are discussed. Furthermore, in order to determine the effects of humidity on the gas sensing properties of the fabricated sensors, the gas sensing measurements were recorded at four different humid conditions, namely at 0%, 25%, 50%, and 75%.

## 2. Materials and Methods

### 2.1. Sensor Preparation

Poly(3-methylthiophene) (PMeT) thin films were deposited on the 10 MHz QCM transducers via electro-polymerization of the monomer 3-methylthiophene (MeT). Electro-polymerization was carried out at room temperature using a CH Instruments Electrochemical workstation system (760 C) with a three-electrode cell using the QCM gold plate as working electrode, a platinum foil as the counter electrode, and an Ag/Ag^+^ electrode as the reference electrode. The potentiostatic method was used to achieve electro-deposition of PMeT. A 50 mM solution of MeT in 0.1 M of lithium perchlorate (LiClO_4_)/Acetonitrile was used to deposit the PMeT film onto the QCM gold plate at 2 V *versus* Ag/Ag^+^ electrode. Thicknesses of the polymer films on the QCM were controlled by the electrochemical process time. In order to investigate the effect of polymerization time and charged-discharged effect on the sensor properties of the polymer films, 5 different samples were prepared denoted here as P1, P2, P3, P4, and P5. P1, P2, and P3 were prepared for 5 s, 10 s, and 15 s, respectively, without a subsequent discharging process. P4 and P5 were prepared for 10 and 15 s, respectively, and these polymer films were subjected to a potential of −0.4 V to entirely discharge the polymer films. The obtained polymer films were rinsed with 2-propanol and dried at room temperature for 1 day before the sensor tests.

### 2.2. Gas Sensing Measurements

In order to investigate the gas sensing properties of the electrochemically coated polymers on the QCM transducers, the sensors were placed into the homemade measurement cell linked to the gas mixing system with the mass flow controller to regulate the composition of the air stream. The frequencies of the chemical sensors were monitored and stored via a data acquisition system controlled by a personal computer. The gas sensing measurements consist of three steps. In the first step, the chemical sensors were exposed to dry air for forty minutes to observe the baseline, and then the actual target analyte at the desired concentration was purged into the measurement cell for twenty minutes. In the third step, the sensors were again exposed to dry air for recovering to the baseline. The second and third steps were repeated four times while increasing the analyte concentrations. All gas sensing measurements were recorded at room temperature (22 °C), and the total gas flow was maintained constant at 200 mL/min. Twelve different analytes including chlorobenzene (100 ppm–500 ppm), n-heptane (390 ppm–1950 ppm), tetrachloroethylene (150 ppm–750 ppm), *o*-xylene (40 ppm–200 ppm), toluene (240 ppm–1200 ppm), ethylbenzene (70 ppm–350 ppm), acetonitrile (950 ppm–4750 ppm), isopropanol (130 ppm–650 ppm), ethylacetate (900 ppm–4500 ppm), triethylamine (720 ppm–3600 ppm), and dimethyl methylphosphonate (DMMP) (3 ppm–15 ppm) were used for determining the gas sensing properties. Furthermore, gas sensing measurements were recorded in various humid air conditions from 25% to 75% r.h. to examine the effects of the humidity on the sensing performance. The details of the experimental gas sensing set-up have been given in previous works [20,21].

## 3. Results and Discussion

### 3.1. Structural Properties

**Figure 1:**
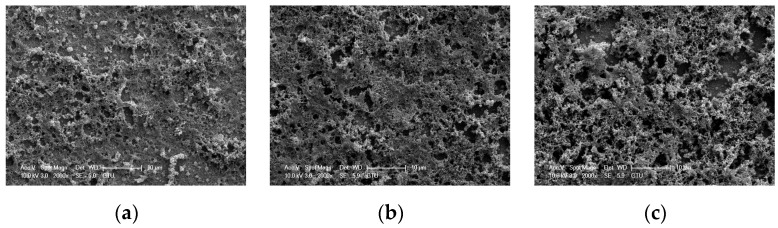
SEM images of the electrochemically deposited polymer thin films on QCM transducers in (**a**) 5 s; (**b**) 10 s; and (**c**) 15 s.

The structural properties of the electrochemically deposited polymer thin films on the QCM transducers were investigated via SEM. The SEM images of the polymers thin films deposited in 5 s, 10 s, and 15 s are given in Figure 1. It can be seen that the surface porosity of the films increased with increasing deposition time. The enhancement of the porosity can be evaluated as increasing the thicknesses of the films. Kayinamura *et al.* investigated effects of the deposition times on the surface porosity of the polymer thin films and discovered that the surface roughness of the films increased with increasing deposition times [22]. Moreover, the thickness of the deposited polymer films on QCM transducers can be controlled in the electrochemical deposition process by varying deposition times. This phenomenon can be observed by measuring the frequency shift of the QCM. Krivan *et al.* investigated mass changes on the QCM due to increasing polymerization times and exhibited that the mass change on the QCM transducers have a linear relationship with deposition time [23].

### 3.2. General Gas Sensing Properties and Discussion of the Sensing Mechanism

**Figure 2:**
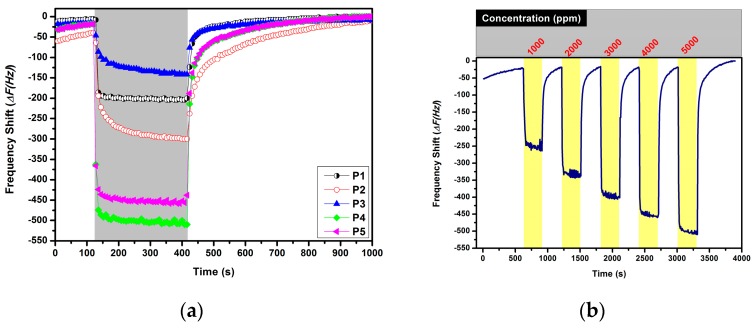
(**a**) Frequency shifts of the PMeT-based QCM sensors P1 to P5 in response to 200 ppm *o*-xylene vapor in dry air (0% r.h.) and (**b**) the time-transient frequency response of P1 during exposure to methanol vapors in increasing concentrations (0% r.h.).

All fabricated sensors are comprised of a conductive polymer layer coated on the mass sensitive transducer QCM. The sensors were tested at room temperature in their responses to various volatile organic compounds including the organophosphorus compound dimethyl methyl phosphonate at various test concentrations to investigate their gas sensing properties, such as sensitivity and selectivity. Figure 2a shows the typical frequency shifts of the QCM transducers modified with polymers deposited under different electrochemical deposition conditions exposed to only one test concentration of *o*-xylene (200 ppm) at room temperature in dry air. It can be seen that the resonance frequency of each sensor decreases very rapidly and then reaches a steady state value. After that, the sensors were exposed to dry air to recover the baseline. Baseline recovery is complete and fast.

It can be observed in Figure 2a that the highest sensor responses were observed for P4 followed by P5 and P2. P1, P2, and P3 were fabricated in the electrochemical deposition process lasting 5 s, 10 s, and 15 s, respectively. The increase of the deposition time improved the doping rates of the PMeT with the ions of the electrolyte [15,17]. The sensor responses of P3 have the highest doping concentration rate decreased drastically and can be attributed to overloading of the QCM transducers with sensitive materials and interrupt sensor responses. Similarly, Si *et al.* showed that the sensing performance of the QCM-based gas sensors decreased with the increasing of the deposition time [4]. Although P4 and P5 were synthesized in 10 s and 15 s, respectively, the samples were discharged under constant negative potential. The discharging of the polymers enhanced the sensor responses compared to the un-discharged samples P1, P2 and P3. The discharging of the polymers is known as de-doping, and doping ions are removed from the chains of the polymers. This process can improve the active sites on the surface [17]. Similarly, the sensor responses of the discharged sensors P4 and P5 decrease with increasing deposition time. In Figure 2b, the time-transient of the resonance frequency of P1 during exposure to methanol while increasing the test concentrations from 1000 ppm to 5000 ppm is depicted. It can be clearly observed that the frequency shift increased depending on the increasing test concentration. Moreover, a shifting of the baseline of P1 was not observed. These typical sensing behaviors were observed for all fabricated chemical sensors for methanol and the other tested analytes including DMMP and humidity. The sensing characteristic of the sensors indicates that the reproducibility of the sensors is quite good for all tested analytes.

Electronic properties of the polymers, especially conductive polymers used as sensitive materials in gas sensing applications, are enhanced/diminished when chemical reactions occur between analyte molecules and the sensitive material. Due to the chemical reactions between the surface of the chemical sensors and the analyte molecules, electrons are transferred from the analyte to the polymers or vice versa; thus, conductivity and other electronic and structural properties of the sensitive materials are altered and can be monitored for detecting gas molecules [24,25]. The chemical reactions between target analytes and sensitive materials based on conductive polymers occur readily at room temperature in case of (reactive) inorganic gas molecules including NH_3_, NO_2_, *etc.* [17]. However, the detection of VOCs is more difficult because VOCs are less reactive and more stable, and electrons may not easily transfer at room temperature [17]. In this aspect, the investigation of the adsorbing mechanism of the gas molecules gains importance. Gas molecules are adsorbed by the surface in two ways, namely, chemical sorption and physical sorption. In the case of chemical sorption, chemical reactions occur between the analyte molecules and surface, and electrons are then transferred. On the other hand, the energy requirement of the chemical sorption is higher than that of physical sorption to take place; therefore, in order to fulfill the energy demand of the chemical reactions, chemical gas sensors either polymer-based or metal oxide-based are operated at elevated temperature, especially for volatile organic compounds [17,26,27]. In case of a physical sorption mechanism, the analyte molecules are adsorbed via weak physical bonding, including van-der Waals forces, dipole-dipole interactions, *etc.* [17,27,28]. Thus, target analyte molecules especially VOCs can be detected easily by monitoring the changes in the physical properties of the polymers as crystal structures or measuring loaded mass on the surfaces via mass sensitive transducers instead of monitoring electronic properties [27,28,29]. Many theories have been published in the literature using adsorption isotherms, diffusion coefficients, and saturated vapor pressure to determine sensing mechanism and surface interactions [17]. Barlett *et al.* produced a model for understanding the sensing mechanism of polymer-based gas sensors by using the Langmuir adsorption isotherm and focused on the occupied and un-occupied sites of the polymer-based sensitive materials [30]. However, there are some missing points in the understanding of the sensing of VOCs by polymers and in the enhancement of the sensing properties of the polymer-based mass sensitive gas sensors. At this point, investigating metal oxide and doped metal oxide gas sensors can be very helpful to clarify VOC detection at room temperature. Generally, metal oxide-based resistive gas sensors are operated at high temperature because the chemical reaction rate is very low at room temperature [27,31]. However, VOCs can be detected by using mass sensitive transducers as the QCM, and the sensing properties (sensitivity, selectivity, etc.) can be enhanced by doping processes or using heterostructures [27]. This phenomenon can be expressed as the following: When gas molecules interact with the metal oxide surface, weak bonds including dipole-dipole interactions or hydrogen bonds occur between the analyte molecules and molecule centers, anionic or cationic atom centers localized on the surface of the metal oxide material, acting as electron accepting and donating sites, respectively [32,33]. Doping or using heterostructures magnifies interaction capabilities of the metal oxide with target analytes and thus sensitivity of the chemical sensors. Similarly, differences in the sensor responses of the polymers due to doping rate can be explained by the increase in the ionicity of the surface, easing the sorption of analyte molecules by the sensitive materials. The VOC molecules can bind to the sensor surface as follows: The bonds of the VOC molecules are cleaved and then dissociated to submolecules groups. These submolecules coupled with the atoms linked to the polymer chains. The activation energy of the VOC molecules is considerably important for bonding on the surface. The de-doping could decrease activation energy between VOCs and sensor surface [15,33,34].

### 3.3. VOC Sensing Performance

**Figure 3:**
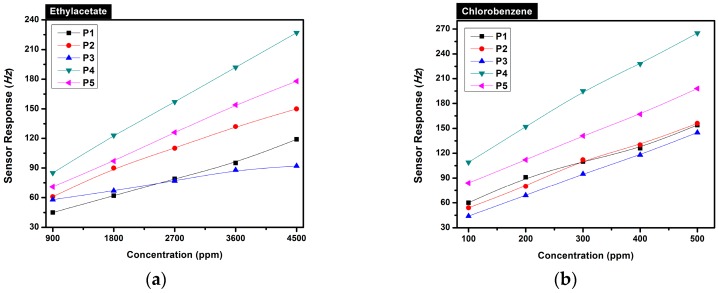
PMeT QCM sensor calibration curves for the analytes in dry air (0% r.h.); (**a**) ethylacetate; and (**b**) chlorobenzene.

Calibration curves of all sensors for the test analytes ethylacetate and chlorobenzene are depicted in Figure 3a,b, respectively. The sensor responses enhance with increasing vapor concentrations, and a linear relationship between concentration and sensor responses was observed. Saturation effects were not seen.

**Figure 4:**
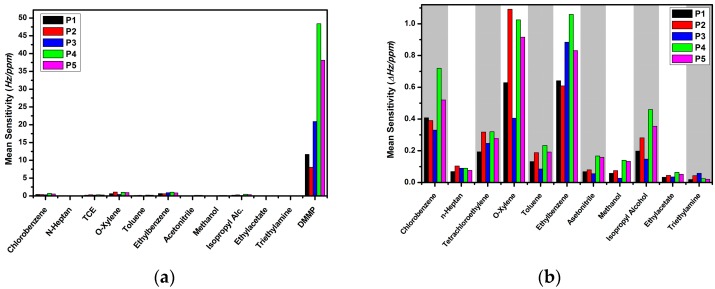
Mean sensitivity in dry air (0% r.h.) of the PMeT sensors for (**a**) all tested analytes and (**b**) all analytes excluding DMMP.

Before investigating effects of the relative humidity on the polymer-based chemical gas sensors, mean sensitivity *S_m_* values of the each sensor and analyte were calculated using Equation (1) [35];

(1) $$S_{m}=\frac{1}{n}\overset{n}{\underset{i}{\sum }}\frac{\Delta f_{i}}{C_{i}}\;\left(Hz/ppm\right)$$

where Δ*f*_i_ is the frequency shift of the QCM transducer while exposed to the desired concentration of the target analyte m, *C_i_* is the concentration value of the target analyte, and n is the number of gas exposures for each analyte. The mean sensitivity calculations make it easier to determine sensor responses and reduce complexity in the gas sensing measurements due to the number of the tested analytes and their test concentrations. The mean sensitivity values of each sensor for all tested analytes are depicted in Figure 4a. It can be clearly seen that the highest sensor responses were observed for DMMP. DMMP is known to be a semi-volatile and sticky compound [20,36] due to the phosphorus atom (P) in the center of the molecular structure. Molecular bonds of DMMP have lower activation energies than those of the other tested analytes, so chemical bonds can be dissociated easily [31]. The activation energy is defined as the required energy for the adsorption of the molecules onto any surface. Wilmsmeyer *et al.* investigated binding energy of the DMMP and dimethyl chlorophosphate onto amorphous silica by using transmission infrared spectroscopy and temperature-programmed desorption to clarify the adsorbing mechanism and found that DMMP needs low energy to adsorb onto amorphous silicium oxide surface, which was as low as 54 kJ/mol. They showed that a main reason for the lower binding energy is dependence on chemical bonds between the P atom and other atoms or chemical groups such as oxygen (O) and methyl groups (CH_3_) [34].

The mean sensitivity values of the sensors for all test analytes except DMMP are plotted in Figure 4b as a bar diagram. The chemical sensors showed the best sensor responses for ethylbenzene and *o*-xylene. The sensor responses against these two mentioned analytes are nearly equal. Both *o*-xylene and ethylbenzene have a CH_3_ group, and the bonding energy to the target analyte molecule is lower than other atoms and molecular groups [28].

The solubility of the polymers or sensitive materials in the presence of the target analyte can be helpful to understand differences in the mean sensitivity values for the tested analytes. Zilbermann *et al.* investigated non-polar volatile organic compound sensing properties of carbon nanotubes/ hexa-peri-hexabenzocoronene in breath and humid air ambient conditions. The sensitive materials were coated on the interdigital electrodes placed on the Si-substrate. The thicknesses of the sensitive materials increased when exposed to the VOC molecules [37]. Moreover, the thicknesses of the sensitive materials were different depending on the target analyte molecules. On the other hand, Ruangchuay *et al.* showed that the length of the polymer chains can expand while exposed to VOCs, and the rate of the expansions is directly linked to the solubility of polymer in target analyte [38].

Limit of detection (LOD) of the sensors and threshold limit values (TLV) for comparison are given in Table 1 [39]. To calculate LOD values, 5 Hz was taken as the lowest detectable signal, and the lowest signal was then divided by mean sensitivity values of each chemical sensor against the tested analytes. Similar calculations were performed in our previous work [21]. The TLV is defined as the exposure limits to VOCs for workers in their daily working life without adverse hazards health effects [40]. The unit of the mean sensitivity is Hz/ppm; LOD and TLV values are provided in ppm. 

The data presented in Table 1 show that the sensitivities of the sensors were enhanced and the LOD values decreased by increasing the deposition time. Furthermore, fabricated samples can be used for monitoring VOC detection indoor/outdoor applications in the absence of relative humidity due to the LOD values of the chemical sensors *versus* the TLV values of the analytes except for triethylamine and acetonitrile.

### 3.4. Responses to Humid Air and Effect on Humidity on VOC Sensing

**Figure 5:**
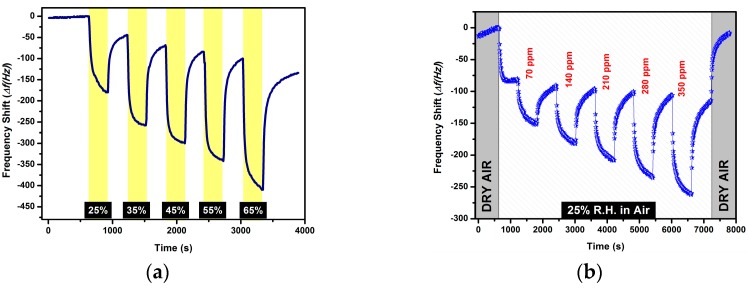

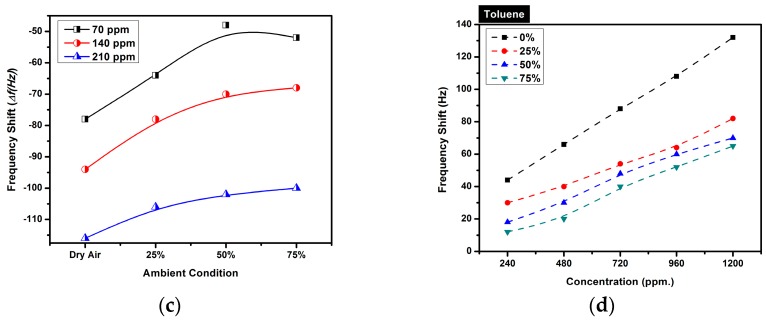
(**a**) Transient signal of the P1 to humid air; (**b**) time-transient of the P1 *versus* ethylbenzene in 25% r.h.; (**c**) sensor responses of the P1 exposed to three test concentrations of ethylbenzene at various test conditions; and (**d**) the calibration curves of the P1 against toluene under varying humidity.

One of the main challenges in chemical gas sensor applications is the presence of humidity in any ambient condition, making the design of technological devices for indoor/outdoor monitoring systems based on laboratory research works difficult. Water molecules can be adsorbed onto the surface of the sensitive materials including not only polymers, but also metals, metal oxides, and organometallic materials, even at room temperature [36]. Adsorption of the water molecules by the sensitive materials often negatively affects their sensing performance in terms of sensitivity, response, and recovery time. Thus, to fully characterize the sensing performance of the PMeT sensors, the gas sensing measurements were recorded in humid air at three different humid air conditions ranging from 25% to 75% r.h. Before the gas sensing measurements in humid air, all QCM sensors were tested to increasing concentrations of water vapor (from 25% to 65% r.h.). The results are depicted in Figure 5a. The frequency shifts of the sensors increased with increasing humidity levels. Although there is a drift in the baseline of the sensors, this can be eliminated by data arrangement algorithms for using real-time monitoring systems. In Figure 5b, the frequency shifts of the chemical sensor during exposure to ethylbenzene vapor of an increasing concentration from 70 ppm to 350 ppm at 25% r.h. humidity in the gas stream is shown. In order to investigate the effects of humidity, firstly dry air was purged into the measurement cell until the sensors reached a stable baseline. Then, the sensors were exposed to humid air (25% r.h.) maintained during the entire duration of the gas sensing measurements. The frequency of the QCM shifted very rapidly, and then reached a steady state value. Afterwards, the QCM-based chemical sensor was exposed to the desired test concentrations of ethylbenzene. The resonance frequency decreased very rapidly after the first analyte exposure. The frequency decrease with increasing concentration can be seen in Figure 5b. Similar results were observed for the other chemical sensors for all other tested analytes, not only at 25% r.h. but also at higher humidity levels. The sensor responses of the chemical sensors are dependent on the humidity level of the air. The sensor responses of the P1 against three different concentrations of ethylbenzene under various ambient conditions (dry air, 25%, 50%, and 75%) are depicted in Figure 5c. The lower sensor responses with increasing humidity in ambient conditions can be attributed to water molecules condensed on the surface covering the whole surface. Covering of the surface causes the number of the active sites to decrease leaving less interaction points between analyte molecules and surface of the sensitive materials. Therefore, increasing humidity in the ambient air has some, albeit limited, negative effects on the gas sensing properties. The calibration curves of P1 against toluene under varying humidity were plotted in Figure 5d; it can be clearly seen that the sensor responses decreased as the relative humidity increases in the air.

## 4. Conclusions

Mass sensitive-based chemical sensors were modified with PMeT via electrochemical deposition while the period of electrochemical deposition and discharging of the sensitive materials were being altered. The fabricated sensor samples were tested in their responses to twelve different analytes including DMMP, which is known as a simulant of G-class warfare agents, at room temperature in dry and humified air. The PMeT QCM sensors showed an at least ten times higher sensitivity to DMMP than to the other tested analytes. The fabricated chemical sensors are a potential candidate for selectively detecting DMMP in ambient conditions. The detection limits of the sensors were lower than TLV values of the tested analytes, except for triethylamine and acetonitrile. Therefore, chemical sensors can be used for the monitoring of the VOCs contaminants in ambient conditions. Moreover, the sensing properties of the fabricated sensors were reduced in case of humid air; however, detection of the analytes was still possible at high humidity levels. As a consequence, PMeT-based chemical mass sensors can be used for detecting various VOCs in any ambient condition and can be used as sensor devices for indoor/outdoor monitoring applications.

## Figures and Tables

**Table 1:** Mean Sensitivity (in Hz/ppm), LOD values (in ppm) of the PMeT-based QCM sensors and TLV (in ppm) of the test analytes.

Analytes/Sensitive Materials	P1		P2		P3		P4		P5		TLV
	*S_m_*	LOD	*S_m_*	LOD	*S_m_*	LOD	*S_m_*	LOD	*S_m_*	LOD	
**Chlorobenzene**	0.4	12.5	0.39	12	0.337	15	0.72	7	0.536	10	10
**n-Heptane**	0.069	72	0.104	48	0.09	55	0.09	55	0.076	66	400
TCE *	0.193	26	0.318	16	0.247	20	0.32	16	0.277	18	25
**o-Xylene**	0.629	8	1.092	4.5	0.405	12	1.025	5	0.916	6	100
**Toluene**	0.132	38	0.188	26	0.085	60	0.233	22	0.192	26	50
**Ethylbenzene**	0.641	8	0.609	8	0.884	6	1.059	5	0.831	6	30
**Acetonitrile**	0.068	75	0.080	62	0.055	90	0.167	30	0.159	30	20
**Methanol**	0.058	86	0.075	66	0.027	185	0.14	36	0.133	38	200
**Isopropanol**	0.198	25	0.281	18	0.147	35	0.46	10	0.354	15	200
**Ethylacetate**	0.033	150	0.045	110	0.035	145	0.064	78	0.053	95	400
**Triethylamine**	0.019	263	0.043	116	0.058	86	0.024	210	0.020	250	1
**DMMP**	11.65	0.42	8.054	0.62	20.90	0.24	48.39	0.1	38.14	0.13	0.1

* Tetrachloroethylene.
